# Does ‘summative’ count? The influence of the awarding of study credits on feedback use and test-taking motivation in medical progress testing

**DOI:** 10.1007/s10459-024-10324-4

**Published:** 2024-03-19

**Authors:** Elise V. van Wijk, Floris M. van Blankenstein, Jeroen Donkers, Roemer J. Janse, Jacqueline Bustraan, Liesbeth G. M. Adelmeijer, Eline A. Dubois, Friedo W. Dekker, Alexandra M. J. Langers

**Affiliations:** 1 Center for Innovation in Medical Education, Leiden University Medical Center, Leiden, The Netherlands; 2 School of Health Professions Education, Faculty of Health, Medicine and Life Sciences, Maastricht University, Maastricht, The Netherlands; 3 Department of Clinical Epidemiology, Leiden University Medical Center, Leiden, The Netherlands; 4 Department of Gastroenterology and Hepatology, Leiden University Medical Center, Albinusdreef 2, 2333 ZA Leiden, The Netherlands

**Keywords:** Progress testing, Feedback use, Formative assessment, Summative assessment, Study credits, Test-taking motivation

## Abstract

Despite the increasing implementation of formative assessment in medical education, its’ effect on learning behaviour remains questionable. This effect may depend on how students value formative, and summative assessments differently. Informed by Expectancy Value Theory, we compared test preparation, feedback use, and test-taking motivation of medical students who either took a purely formative progress test (*formative* PT-group) or a progress test that yielded study credits (*summative* PT-group). In a mixed-methods study design, we triangulated quantitative questionnaire data (*n* = 264), logging data of an online PT feedback system (*n* = 618), and qualitative interview data (*n* = 21) to compare feedback use, and test-taking motivation between the *formative* PT-group (*n* = 316), and the *summative* PT-group (*n* = 302). Self-reported, and actual feedback consultation was higher in the *summative* PT-group. Test preparation, and active feedback use were relatively low and similar in both groups. Both quantitative, and qualitative results showed that the motivation to prepare and consult feedback relates to how students value the assessment. In the interview data, a link could be made with goal orientation theory, as performance-oriented students perceived the *formative* PT as not important due to the lack of study credits. This led to low test-taking effort, and feedback consultation after the *formative* PT. In contrast, learning-oriented students valued the *formative* PT, and used it for self-study or self-assessment to gain feedback. Our results indicate that most students are less motivated to put effort in the test, and use feedback when there are no direct consequences. A supportive assessment environment that emphasizes recognition of the value of formative testing is required to motivate students to use feedback for learning.

**Supplementary Information:**

The online version contains supplementary material available at 10.1007/s10459-024-10324-4.

## Introduction

The notion that ‘*assessment drives learning’* is widely acknowledged (Al-Kadri et al., 2012; Newble & Entwistle, 1986). The way learning is driven may therefore differ with the focus of the assessment. Within medical education, the focus is mainly on assessment *of* learning (Schuwirth & Van der Vleuten, 2011). These summative assessments evaluate students’ learning by measuring their performance, often reported as a summative grade. In contrast to assessment *of* learning, assessment *for* learning uses formative assessments to enhance in-depth learning, and self-regulated learning (SRL) by providing ongoing feedback (Berkhout et al., 2018; Black & Wiliam, 1998; Brown et al., 2016; Castro et al., 2021; Koh, 2008; Kulasegaram & Rangachari, 2018; Schuwirth & van der Vleuten, 2012; Scott, 2020; Seligman et al., 2021; Watling & Ginsburg, 2019). More specifically, formative feedback provides opportunities for reflection, identifying learning gaps, and adjusting learning, which are important aspects of SRL (Black & Wiliam, 1998; Hattie & Timperley, 2007; Zimmerman, 2008). In this way, feedback can also stimulate the use of learning strategies that enhance future learning performance (Nicol & Macfarlane-Dick, 2006). With the growing consensus that assessment should promote learning, and in light of these positive learning effects, there is a shift in assessment *of* learning towards assessments *for* learning in medical education (Schuwirth & Van der Vleuten, 2011; Scott, 2020). However, to facilitate this shift, further elucidation of the complex relationship between assessment, learning,and the driving factors behind students’ learning is needed.

One of the factors found to drive students’ motivation to learn is increasing the weight of summative assessments (Wormald et al., 2009). Motivation to learn for an assessment also affects test-taking motivation: students’ readiness to invest effort in a test (Baumert & Demmrich, 2001; Cole et al., 2008; Thelk et al., 2009; Wise & DeMars, 2005). Considering the lack of direct consequences of formative test results, students might be less motivated to put their best effort in these tests. This can be explained by the Expectancy-Value Theory (EVT), a conceptual framework frequently used in the context of test-taking motivation. This theory assumes that motivation for a task depends on expectancies of success, and perceived value given to the task (Eccles & Wigfield, 2020). Specifically, motivation for a task increases when people expect to be successful and when they find the task valuable for themselves. Test-taking effort is the main element of test-taking motivation, which, according to the EVT, is thus the direct outcome of expectancy and value. Most studies that investigated EVT in the context of test-taking motivation focused on the value component of EVT. Overall, these studies report positive relationships between value and test performance, and also between test effort and test performance (Cole et al., 2008; Zilberberg et al., 2014).

Another way to look at *‘assessment drives learning’* is through the lens of the goal orientation theory. This theory states that the individual goal orientation affects motivation, which in turn guides behavioural responses (Elliot & Dweck, 2005). Goal orientation can either rely on learning (mastery- or learning-oriented goals) or performance (performance-oriented goals). Learning-oriented students might take a different approach in making a test, and using its feedback than performance-oriented students, but so far the influence of goal orientation in different assessment conditions has not been investigated.

One way to investigate the differences between different assessment conditions is by using the medical progress test (PT), which is a frequently used assessment method in medical education. The PT is a longitudinal, comprehensive, and curriculum-independent test administered repeatedly to assess students’ knowledge progress and provide feedback(Tio et al., 2016; Van Der Vleuten et al., 1996). The PT combines longitudinal testing with feedback, serving an important formative function, but in many educational contexts the results of PTs are also used for a summative pass/fail decision followed by the rewarding of study credits. As the PT covers the entire medical curriculum, it discourages test-directed studying, and encourages self-directed learning by using the feedback of the previous PT (Van Der Vleuten et al., 1996). Implementing frequent PTs with a summative component, and the integrating purely formative PTs (no study credits involved) in a curriculum with other formative assessments has shown a positive impact on students’ test-effort, perceived learning value, and feedback use (Dijksterhuis et al., 2013; Heeneman et al., 2017; Norman et al., 2010; Schüttpelz-Brauns et al., 2020; Wade et al., 2012). However, some studies have not found the expected beneficial impact of feedback in purely formative PTs on learning (Aarts et al., 2010; Given et al., 2016; Van Der Vleuten et al., 1996; Wrigley et al., 2012; Yielder et al., 2017). Different educational conditions affect the test-taking effort, and the perceived value of purely formative PTs (Schüttpelz-Brauns et al., 2020). These PTs have no direct consequences (i.e. no ‘stakes’) for study progress, which may lead to a lower perceived value, which in turn may result in less test-taking motivation and effort put in these tests (Barry et al., 2010; Schüttpelz-Brauns et al., 2020). Besides an impact on test performance, this might also affect their use of feedback.

In summary, while assessment should promote learning (i.e. assessment *for* learning), the actual effect of formative assessments on learning is unclear. More specifically, it remains unclear how formative versus summative assessment affects students’ feedback use, and test-taking motivation. The PT provides a unique opportunity to study this distinction, especially when we can compare a purely formative PT with a PT that also has a summative component. Understanding how students adapt their learning behaviour to formative versus summative assessment may help teachers optimize both functions of assessment, as it enables them to react to the student’s behaviour in order to promote their learning process, and foster lifelong learning. Therefore, we aimed to investigate the effect of a PT with a summative component (*summative* PT), and a purely formative PT (*formative* PT) on medical students’ (1) test preparation, (2) factors that influence test taking motivation, and the use of feedback, and (3) self-reported, and actual feedback use after the test.

## Methods

### Study design

We used a convergent mixed-methods approach with a subtle realism paradigm, involving a questionnaire, online Progress test Feedback system (ProF) logging data, and semi-structured interviews. The subtle realism paradigm combines a realist ontology (an objective reality independent of our perceptions) with a constructivist epistemology (our understanding of reality depends on our perspectives) (Fetters et al., 2013; Maxwell & Mittapalli, 2010). This paradigm aims at representing reality rather than attaining ‘*the truth’*, by triangulating different data sources, perspectives, and theories. We chose this approach as this best aligns with our research design,which attempts to represent, and deepen our understanding of reality (‘feedback use in the context of different assessment conditions’) by the triangulating different data sources, and theories. This paradigm allows us to integrate different perspectives while remaining flexible in interpreting our qualitative data. All data types were analysed separately and converged in a final interpretation phase, where we compared the results of the quantitative and qualitative data, and assessedwhether the data confirmed or disconfirmed each other. Our qualitative results, using the existing theoretical frameworks of EVT and goal-orientation theory, helped us understand, and explain the observed and self-reported quantitative feedback behaviour.

### Setting

The study was conducted at Leiden University Medical Center (LUMC) in the Netherlands. The medical curriculum in the Netherlands includes a three year preclinical Bachelor program and three year clinical Master program. The Bachelor program comprises several theoretical courses,assessed by written summative assessments at the end of each course, and rewarded with study credits. Most courses also offer a formative assessment for practice, which is not mandatory for students to take. In the Master program, students undergo clinical rotations, assessed by a pass or fail decision based on supervisor feedback. Throughout their six years of medical school, all medical students take four PTs per year, resulting in a total of 24 test moments (Tio et al., 2016). The PTs are taken in September (PT 1), December (PT 2), February (PT 3), and May (PT 4). The PT is a comprehensive written testof 200 multiple choice questions (MCQs), covering all relevant medical disciplines, and stratified in categories (Van Der Vleuten et al., 1996). The MCQs include a question mark option that yields no points, and points are deducted for incorrect answers (Lord, 1975). All participating students take an identical PT in an exam hall with proctoring. The final score on the PT is expressed as a percentage of the maximum attainable score, which is translated into “*Good”, “Pass”*, or *“Fail”*, based on the mean, and standard deviation of the students that participated in the same test moment as a relative standard. The scores of the four PTs in every academic year are combined, and translated into a summative decision, followed by the awarding of two study credits (of the in total 60).

After each PT, students can check their answers with an online answer key. For each answer a source is provided for further information, and for some answers a short explanation is given. Additionally, students receive their score and feedback via e-mail (Online Resource 1), and they can access feedback in ProF in the form of a table displaying their individual score, stratified by category and discipline, compared to the overall score of their peers. In ProF, their individual longitudinal test results are visualized in graphs as well (Tio et al., 2016). There is no option to download the feedback displayed in ProF. Students receive information about the PT, and the use of ProF through a lecture in each of the bachelor years. Reflection on the feedback with their supervisor is optional.

**Fig. 1:**
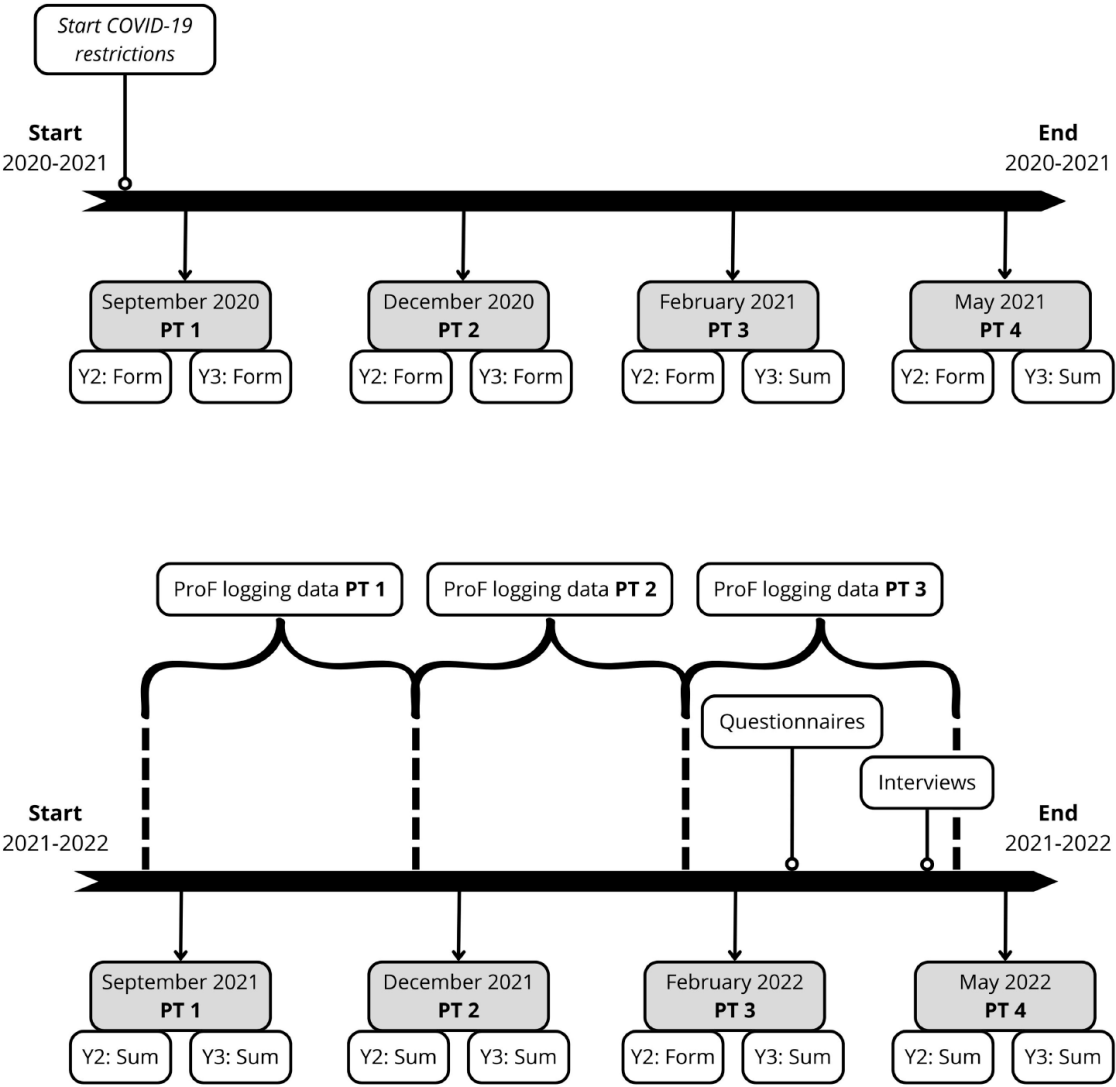
Timeline of the progress tests and associated data collection during the academic year 2020–2021 (top) and 2021–2022. PT = progress tests; Y2 = second-year students; Y3 = third-year students; Form = formative; Sum = summative. In 2021–2022, PT3 was purely formative for Y2 students because there was no access to online proctoring, and summative for Y3 students

Due to the COVID-19 restrictions, some of the PTs in the LUMC during the academic years 2020–2021 and 2021–2022 were taken from home by students, via a digital assessment platform. As the COVID-19 restrictions intensified during the pandemic (e.g. total lockdown), exam conditions varied as well. Some of the online PTs used online proctoring software, and were summative (e.g., PT1 and PT2 in 2021–2022). However, in February 2021 (PT3), we could not access the online proctoring system due to logistic reasons. Part of the students could take the PT in the exam hall, but its capacity was largely reduced due to COVID-19 regulations. As a result, the exam hall could only harbour one cohort, i.e. the third-year students. Second-year students took the PT from home, online and non-proctored. As a result, the PT was *summative* for third-year students, and *formative* for second-year students. Figure 1 shows which PTs were *formative*, and *summative* for these two cohorts. We show the situation for these two cohorts only, because these cohorts are our main focus. Students were instructed to take the *formative* PT as a usual (proctored) PT, without using study materials, but without proctoring, we could not verify if students followed these instructions. Participation in these PTs was mandatory, but the test results were not taken into account for the rewarding of study credits. Therefore, these non-proctored PTs turned into purely formative assessments. Hereafter we will call this PT the *formative* PT, whereas the proctored PT that counts towards study credits will be called the *summative* PT.

### Participants

All second-, and third-year bachelor medical students at the LUMC who participated in the PT session on February 2, 2022 (PT 3 of 2021–2022), were eligible for participation in the questionnaire part of the study, and all second- through six-year medical students at the LUMC were eligible for the interviews. The PT session on the February, 2, 2022 was purely formative (*formative* PT) for second-year students, while the result of the PT was taken into account for study credits in third-year students (*summative* PT). Inclusion criteria for semi-structured interviews were (1) participation in at least four of the six PTs between September 2020, and December 2021, and (2) participation in both a *formative* and *summative* PT. In total, 1286 students met our inclusion criteria. Students were sampled using maximum variation sampling based on ProF logging sessions, study-year, and PT results to ensure the representation of multiple perspectives (Onwuegbuzie & Leech, 2007). Sampling of these students was informed by quantitative data, such as ProF logging sessions and study-year. The groups for the number of ProF logging sessions were based on the distribution among all students who participated in the PTs. The PT results were divided in two groups: “*fail*” or “*pass/good*”. If a student had failed on at least one PT, the student was assigned to the “*fail*” group (*n* = 410). The other students were assigned to the “*pass/good”* group (*n =* 876). We initially approached 140 students that met our sampling strategy, of whom 18 were interested. The distribution of these students was a good representation of our sampling groups, so we invited all 18 students for an interview. After this initial sampling, second-year students were still underrepresented compared to third-year students, so we decided to sample and approach additional second-year students. Three students replied, who were all included in our study. In total, this resulted in 21 interviews, and a more equal distribution among second- (*n* = 6) and third-year students (*n* = 8).

### Data collection

#### Questionnaire and ProF logging data

A questionnaire was completed either digitally or on paper (Online Resource 2). It measured perceived assessment condition, test preparation, feedback consultation, and active use of feedback. *Perceived assessment condition* was measured with two MCQs (‘formative, summative or don’t know’, and ‘high, intermediate or low stakes’). These items were added to compare true assessment conditions with perceived assessment conditions. *Preparation* and *feedback consultation after the PT* were measured with two yes/no questions. Students were also asked to select explanatory reasons for their answers. *Active feedback use* was measured with the Active Use of Feedback (AUF) scale. This scale consists of seven 6-point, positively packed Likert-items, and is part of the validated, revised version of the Students Conceptions of Feedback (SCoF) Questionnaire (Brown et al., 2016). Six of the seven original AUF scale items, and one item of the ‘*Enjoyment’* (ENJ) subscale were used. The items were adapted to the context of the PT (e.g., ‘tutor’ was replaced by ‘progress test’). Two items were excluded because they did not apply to the specific context or were very similar to another item. The items were translated to Dutch using a forward-backward translation method. The content, and structure of the questionnaire were assessed by three master students using a thinking aloud method. Two weeks after the PT scores and feedback were made available, students received the digital questionnaire by e-mail. We also visited lectures, and working groups to hand out paper questionnaires. The students received up to two digital reminders. Age, PT grades, and ProF logging data of all students (both responders and non-responders of the questionnaire) were derived from the university’s student administration system.

#### Interviews

We developed an interview guide to explore which factors affect feedback use in progress testing (Online Resource 3). The interview data were part of a more comprehensive study on factors influencing feedback use in progress testing (van Wijk, 2023). In this study, we only selected interview data about students’ perceptions of feedback use in the context of a *formative* and *summative* PT. Besides their own perceptions, we asked students to reflect on the ProF logging data from all bachelor, and master students in relation to *formative* and *summative* PTs during the COVID-19 pandemic (Online Resource 4).

The principal investigator (EvW) conducted two pilot interviews with fourth-year medical students, which resulted in minor revisions in the interview guide to improve clarity and structure. The pilot interviews were not included in the study. EvW conducted the interviews with 21 students (Online Resource 5) via online meetings in Microsoft Teams in April and May 2022. Participants were invited by e-mail, and received an electronic gift card in return for participation. The interviews took 30–60 min, and were audiotaped. The audiotapes were transcribed verbatim, and anonymized before analysis. The timeline of the data collection from the different sources are depicted in Fig. 1.

### Data analysis

#### Questionnaire

Descriptive statistics were calculated for the demographics, and perceived assessment conditions. Standardized mean differences (SMD) (Austin, 2011) were calculated to quantify baseline group differences between the *formative*, and *summative* PT-groups, and to explore potential response bias (non-responders versus responders). Logistic regression analyses were used to study the effect of assessment condition on test preparation, and feedback consultation. Cronbach’s α was calculated to assess internal consistency of the AUF scale items. Differences between the *formative*, and *summative* PT-group were assessed by an unpaired t-test (total mean score on the AUF scale items), and chi-squared tests (multiple-choice questions on preparation, and feedback consultation). Subgroup analyses were performed on students from whom the perceived assessment condition (formative or summative) matched the actual assessment condition. We used the actual assessment conditions for our main analyses, because students were well aware of the physical difference in tests condition (i.e., from home without any webcam observation versus in an exam hall with continuous supervision), and therefore we assumed that this would be a more important discriminative factor than the perceived formative or summative test condition.

#### ProF logging data

All ProF sessions were included for analysis, independent of the number of pageviews or duration of their session. The average number of ProF sessions per student was calculated for the PTs in September 2021 (PT 1), December 2021 (PT 2), and February 2022 (PT 3). We chose a time range of one week before the PT until one week before the subsequent PT to assess both feedback consultation before (for preparation) and after the PT. Linear regression was used to estimate the effect of assessment condition on average number of ProF sessions for the PT in February 2022, adjusted for ProF-sessions on previous PTs (December 2022, and September 2021). Adjustment for ProF-sessions in December 2022, and September 2021 was done by adding the number of ProF-sessions around these PTs as two separate covariates in our linear regression formula. To cross-check the self-reported ProF consultation after the PT on the questionnaire, we analysed the ProF logging data of the responders in the week of the PT in February 2022 until the end of the questionnaire administration (6 weeks later). For both the questionnaire, and ProF logging data analysis, statistical significance was determined by a 95% confidence interval (CI) and *p* < 0.05. Data were analysed using R version 4.1.0 (R Foundation for Statistical Computing, Vienna, Austria).

#### Interviews

Data analysis started after four interviews, which led to small adjustments in the interview guide to specify the questions more. The remainder of the data analysis took place after all interviews were completed. Because the extensive literature on feedback use can be integrated by *a priori* themes that guide the deductive analysis, we used template analysis in which hierarchical coding and development of successive coding templates is used (Brooks et al., 2015). Our *a priori* themes were based on EVT in the context of TTM (Baumert & Demmrich, 2001; Cole et al., 2008; Eccles & Wigfield, 2020). Two independent coders (EvW and FvB) coded interviews 1–6 in Atlas.ti. This was discussed afterwards together and with a third researcher (AL) to reach consensus on the initial template, which was then used to guide the coding of the next interviews. Analysis of interviews 7–14 was used to further revise the initial template (EvW and FvB) which in turn was used to code interviews 15–21, and develop the final template. Only minor revisions were made to the revised initial template, and no new themes related to the research question raised in the development of the final template, indicating theoretical sufficiency after interview 14 (Dey, 1999; Saunders et al., 2018). The final template was discussed with the research team (EvW, FvB, AL, JB). During the iterative process, elements of the EVT and goal-orientation theory were incorporated in the template. Eventually, EvW reread, and recoded all interviews with the final template to ensure all relevant information to answer the research question was included in the template. With this final template, a thematic-map was constructed to identify connections between the themes and codes. Member checking was done using the Synthesized Member Checking (SMC) method (Birt et al., 2016), and yielded no adjustments.

#### Reflexivity

We considered and discussed (inter)personal reflexivity throughout our data collection, and analysis process using a reflective diary and critical dialogues regarding our interpretations of the data (Olmos-Vega et al., 2022). The reflective diaries created awareness of personal expectations, assumptions, and reactions to the participants and data, and were used to guide the dialogues between the investigators. In interviewing the students, EvW experienced that she could easily relate to the participants, because of her own medical background and experience with the PT. This created an open atmosphere, in which the students felt comfortable to talk openly about their experiences and perceptions. Influenced by her scientific background in (bio)medicine, EvW attempted to attain as much objectivity and produce rigorous qualitative research by using maximum variation sampling, member checking, and reflexivity throughout the data collection and analysis. The other researchers were an educational consultant and researcher in medical education (FvB) and a medical doctor with experience in clinical teaching and educational research (AL). FvB has been trained to conduct research in an empirical way during his studies in cognitive psychology. As such, he supported using theoretical concepts from feedback literature to formulate *a priori* themes. This theory-driven approach may have influenced the results. AL is a member of the national PT working group and a PT examiner, which might have led to assumptions on study behaviour based on her experience with the PT and conversations with students in the past. Her involvement with the PT was very valuable in reflecting on the interview data, and placing it in the right context.

## Results

### Demographics and perceived assessment condition

Of 316 students who took the purely formative PT (*formative* PT-group), 113 students participated in the questionnaire (response rate: 35.8%). In the *summative PT*-group, 154 students participated in the questionnaire (response rate: 50.0%) from which 3 students were excluded due to incomplete reply to the questionnaire (Online Resource 6). Responders (*n* = 264) and non-responders (*n* = 354) differed in fail/pass/good grade and average ProF logging sessions (mean (SD); 1.29 (1.60) versus 0.71 (1.87), for responders versus non-responders) (Online Resource 7 ). In both the *formative* and *summative* PT-group, 70% of the responders were female, and the distribution of the grades was similar among the groups (Table 1). Regarding the perceived stakes of the PT in February 2022, 50% versus 13% of students perceived the PT as low stakes, 42% versus 62% as intermediate stakes, and 7% versus 25% as high stakes for *formative* and *summative* PT-group respectively (Online Resource 8). The perceived assessment conditions formative and summative can also be found in Online Resource 8.

**Table 1:** Baseline characteristics of the responders of the questionnaire in the *formative* and *summative* progress test-group

	Overall (*n =* 264)	Formative Test (*n =* 113)	Summative Test (*n* = 151)	SMD^a^
Age, median (IQR)	21 (20, 21)	20 (20, 21)	21 (21, 22)	0.841
Female, n (%)	185 (70)	80 (71)	105 (70)	0.022
Grade, n (%)				
Fail	24 (9)	11 (10)	13 (9)	0.034
Pass	106 (40)	46 (41)	610 (40)	0.020
Good	134 (51)	56 (50)	78 (51)	0.040
Proportion passed earlier PTs, %				
Sep ’21^b^	223 (87)	99 (88)	124 (87)	0.030
Dec ’21	226 (87)	97 (86)	129 (88)	0.059

SMD, standardized mean difference; IQR, interquartile range; PT, progress test

^a^ A standardized mean difference > 0.1 may point towards meaningful imbalance between groups

^b^ PTs of Sep ’21 and Dec ’21 were summative tests

In the following paragraphs we present the results for each research question: the effect of a *summative* PT and a *formative* PT on medical students’ (1) test preparation (questionnaires, and interviews), (2) factors that influence test-taking motivation, and the use of feedback (interviews), and (3) self-reported and actual feedback use after the test (questionnaires and ProF logging data, and interviews).

#### Test preparation

Logistic regression showed no significant association between assessment condition and preparation for the PT (adjusted OR [aOR] 1.26, 95% CI 0.57–2.76) (Table 2). A similar result was found in the subgroup analysis (aOR 1.83, 95% CI 0.72–4.64).

**Table 2:** Test preparation, feedback consultation and active use of feedback of students in the *formative* and *summative* progress test-group

	Formative Test	Summative Test	Crude OR (95% CI)	Adjusted OR (95% CI)^a^	p-value
True formative and summative					
*Number of individuals*	113	151			
**Preparation**, n (%)	14 (12)	28 (19)	1.61 (0.80–3.22)	1.26 (0.57–2.76)	0.568
**Feedback consultation**, n (%)					
Answer key	22 (19)	56 (37)	2.44 (1.38–4.32)	1.92 (1.04–3.55)	0.038
Feedback e-mail	89 (79)	126 (83)	1.36 (0.73–2.53)	1.00 (0.49–2.05)	0.996
Feedback ProF	41 (36)	86 (57)	2.32 (1.41–3.83)	1.92 (1.10–3.34)	0.021
None	20 (18)	13 (9)	2.28 (1.08–4.81)	1.86 (0.80–4.32)	0.149
**ProF logging data**^c^, n (%)	26 (23)	58 (38)	2.09 (1.21–3.61)	1.89 (1.03–3.44)	0.039
*Number of individuals* ^*d*^	90	135	**t-value**	**95% CI**	
**Active use of feedback**, Mean (SD)	3.2 (0.9)	3.1 (0.9)	1.09	-0.10-0.36	0.275^e^
**Perceived formative and summative** ^**b**^					
*Number of individuals*	79	128			
**Preparation**, n (%)	8 (10)	25 (20)	2.15 (0.92–5.05)	1.83 (0.72–4.64)	0.205
**Feedback consultation**, n (%)					
Answer key	18 (23)	48 (37)	2.03 (1.08–3.84)	1.47 (0.73–2.94)	0.280
Feedback e-mail	60 (76)	106 (82)	1.53 (0.76–3.04)	1.07 (0.48–2.39)	0.876
Feedback ProF	26 (33)	74 (57)	2.79 (1.55–5.02)	2.25 (1.18–4.31)	0.014
None	15 (19)	11 (9)	2.49 (1.08–5.75)	1.94 (0.74–5.05)	0.175
**ProF logging data**^**c**^, n (%)	17 (22)	48 (38)	2.19 (1.15–4.17)	1.80 (0.88–3.66)	0.106
*Number of individuals* ^*d*^	62	114	**t-value**	**95% CI**	
**Active use of feedback**, Mean (SD)	3.2 (0.8)	3.1 (0.8)	0.94	-0.14-0.38	0.351^e^

^a^ Adjusted for age and result progress test dec ‘21 (fail, pass, good)

^b^ Subgroup analysis; Perceived formative/summative: students in the purely formative/summative test group who knew it was formative/summative

^c^ Real-time ProF logging data in week 05 (PT administration) until week 11 (end of questionnaire administration)

^d^ Students who consulted feedback in e-mail or progress test feedback system

^e^ Unpaired t-test

Regarding the reasons why students did not prepare for the PT, 27% of the students in the *formative* PT-group stated on the questionnaire that the PT was not important compared to 1% of the students in the *summative* PT-group (*p* < 0.001, Online Resource 9). In the subgroup analysis this difference became more prominent (26 (37%) versus 0 (0%), *p* < 0.001 for *perceived formative* versus *perceived summative*). Other reasons for not preparing were a lack of consequences and not knowing how to prepare.

In the interviews, many students mentioned that the lack of consequences and the possibility to look up answers in the *formative PT* affected their test preparation:

"My preparation for a formative PT is worse. I still look up some things in advance which I just want to know, but there is less pressure, so if it does not work out or if I don’t really feel like doing it, then I think, well, if a question comes up I don’t know, I can just look it up.” (Interview #3).

#### Factors that influence test-taking motivation and feedback use

**Fig. 2:**
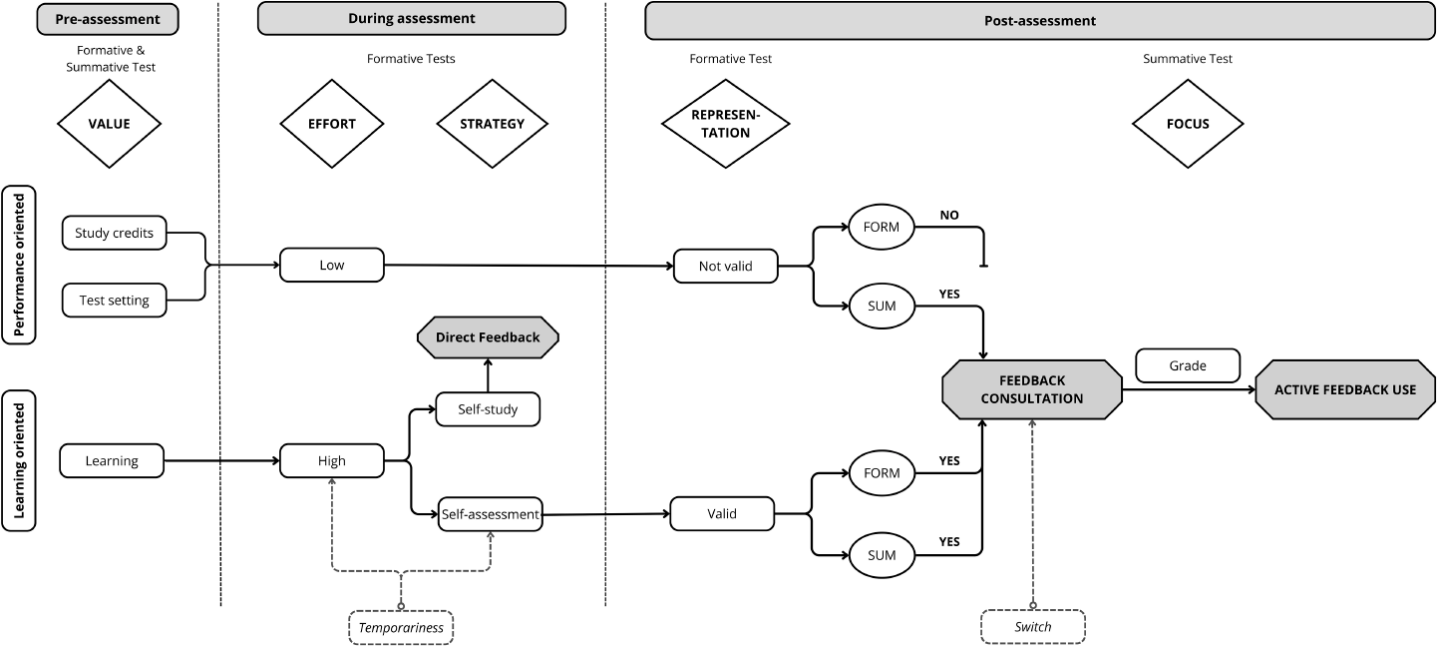
Thematic map showing the connections between the themes (triangular shapes on the top) and codes (boxes below the themes) pre-, during and post-assessment for performance-oriented (upper path) and learning-oriented students (lower path). Form: formative progress test; Sum: summative progress test

The **value** given to the *formative* and *summative* PT influenced students test-taking motivation, and determined how students behaved during the *formative* PT (i.e., test-taking behaviour). The majority of students valued the *summative* PT as more important compared to the *formative* PT, because of its’ consequences for study progress, and the more formal test-setting compared to the *formative* PT (on location vs. at home). We call these students ‘**performance-oriented**’ (Fig. 2, upper path):

"Ultimately, you take each test for the study credits. You follow the lessons to learn something, but I do not make a test to learn from it. I make a test to see if my learning was successful. And whether or not I can receive the credits so I can continue.” (Interview #4).

On the other hand, ‘**learning-oriented**’ students valued the test and its’ feedback as a moment of self-assessment and reflection, regardless of the assessment condition. Their main focus in both the *formative* and *summative* PT was to assess their current knowledge level, gain insights in their own strengths and weaknesses and learn from what they did wrong (Fig. 2, lower path).

#### Test-taking behaviour: effort and strategy

We distinguished two subthemes within test-taking behaviour: **effort** and **strategy**. These themes only relate to the *formative* PT, because the low-stakes and lack of supervision were perceived as an opportunity to adapt their test-taking behaviour according to their values and goals in relation to the PT.

Learning-oriented students tended to put significant effort in the *formative* PT, as they wanted to be able to reflect effectively on their performance. In contrast, performance-oriented students put less effort in taking the *formative* PT, reflected by a higher proportion of guessing, looking up answers on the internet or being less focused during the test:

"I think that I guessed more of the answers in the online [formative] test when I recognized an answer vaguely from a previous course. I did not know the answer completely for sure, but I was doubting between three options and then I just guessed because it did not matter so much.” (Interview #7).

Students employed different test-taking **strategies** in the *formative* PT, which could be divided in self-study and self-assessment. The self-study strategy was characterized by using study materials to look up answers during the test, mainly with the idea to learn directly from it. By looking up answers for questions, they generated instant feedback for themselves and hence used the *formative* test as a guide for self-study:

"Well, I thought if I look it up right away I will learn something from it, because then I know the answer. And if I will not look at it anymore afterwards, then I actually do not learn so much either, because I don’t know if my answers were correct or incorrect.” (Interview #9).

In the self-assessment strategy, students approached the *formative* PT as if it were a *summative* PT and refrained from looking up answers. They used the test as a realistic self-assessment of their current knowledge:

"When you get the result, that you have some sort of measurement of how good you actually are at it. Because otherwise [when using study material] I have the idea that it does not make sense at all to take that test.” (Interview #10).

#### Contextual factors: Temporariness

Many students took into account that the *formative* PTs were only temporary and that in the near future, they would become summative again. This temporariness encouraged them to make the *formative* test just as seriously as the *summative* test, with an indirect focus on study credits relating to the performance-oriented mindset:

"Of course I could have looked it all up, but then I think you will fall at a certain moment. I think you cannot sustain that when the test is proctored again. And then it’s only annoying that you’re going to drop in your score again.” (#Interview 14).

### Self-reported and actual feedback use after the test

#### Feedback consultation

**Fig. 3:**
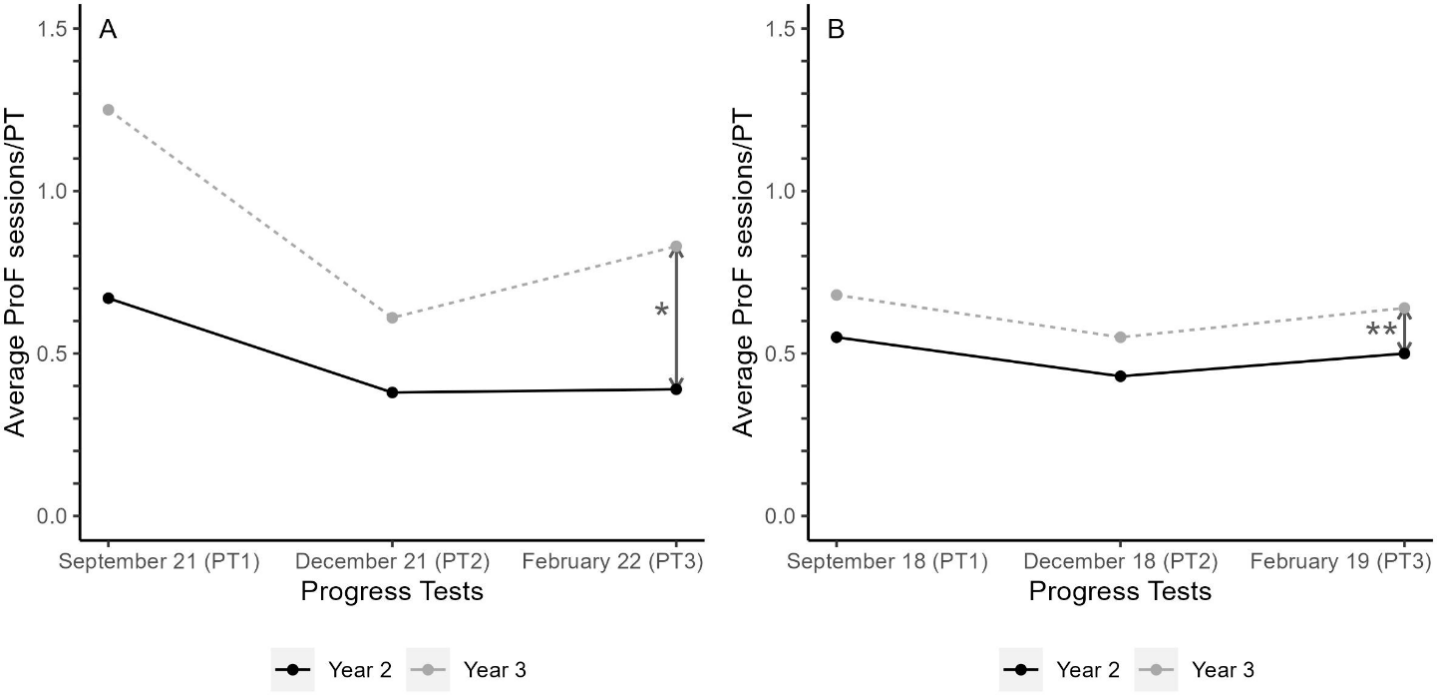
Average ProF sessions in year 2 (black line) and year 3 (dotted grey line) for the progress tests in September 2021, December 2021, and February 2022. Each point on the curve represents the average number of ProF sessions per student; *: crude β:0.444, p:<0.001; adjusted β:0.251, p:0.003

Students who took the *summative* PT reported consulting ProF (aOR 1.92, 95% CI 1.10–3.34) and the answer key (aOR 1.92, 95% CI 1.04–3.55) more often than students who made the *formative* PT. In *perceived formative* versus *summative*, the effect on feedback consultation in ProF became more evident (aOR 2.25, 95% CI 1.18–4.31) and the adjusted effect on the answer key consultation was not observed (aOR 1.47, 95% CI 0.73–2.94) (Table 2). The sensitivity analysis with real-time ProF logging data of the responders showed the same trend as the self-reported data, but yielded lower overall numbers (26 (23%) versus 58 (38%), aOR 1.89, 95% CI 1.03–3.44; for *formative* versus *summative*; 17 (22%) versus 48 (38%), aOR 1.80 (0.88–3.66); for *perceived formative* versus *perceived summative*) (Table 2). Besides the sensitivity analysis using only ProF logging data of the responders, we also analysed the ProF logging data of all participating students including the non-responders. This analysis showed that there were more ProF logging sessions around the *summative* PT in February 2022 than around the *formative* PT (β:0.444, p:<0.001). After adjustment for logging behaviour in earlier *summative* PTs in September 2021 and December 2022, this effect remained significant (β:0.251, p:0.003) (Fig. 3, Online Resource 10).

Students who reported not to consult the feedback after the *formative* PT on the questionnaire more often considered the test as not important (15 (22%) versus 1 (2%), p: <0.001 for *formative* versus *summative*) (Table 3). This resonates with the perceptions of the performance-oriented students:

I think that you are more motivated when the test counts for study credits, [.] so then afterwards you will be more interested in how you performed because it counts.” (Interview #10).

However, qualitative data also revealed the learning-oriented students who valued the feedback of both assessment conditions for their learning (Fig. 2, lower path):

"I look at the test result to know what questions I did wrong and to learn from it. And it doesn’t matter to me whether it is formative or summative, because that remains the same. I still want to know which questions I got right and wrong. And I still want to know, I still want to learn from the things I did wrong. So, then it doesn’t matter if the test was formative or summative.” (Interview #3).

In the questionnaires, the *summative* PT-group more often found the feedback not useful (1 (1%) versus 8 (13%), p: 0.015 for *formative* versus *summative*). Similar results were found for perceived assessment conditions (Table 3). Other reasons for not consulting feedback included no awareness of or not understanding ProF, not knowing how to use the feedback, and a lack of interest.

**Table 3:** Reasons for not using the progress test feedback system in the formative and summative progress test group

	Formative Test	Summative Test	p-value^a^
True formative and summative			
*Number of individuals*	67	63	
**No ProF use**, n (%)			
Findability^c^	25 (37)	14 (22)	0.061
Time	24 (36)	16 (25)	0.178
Effort	7 (10)	1 (2)	0.062^d^
Importance	15 (22)	1 (2)	0.000
Grade	33 (49)	22 (35)	0.083
Utility	1 (1)	8 (13)	0.015^d^
Answer key	3 (4)	7 (11)	0.200^d^
Other	4 (6)	12 (19)	0.025
**Perceived formative and summative** ^**b**^			
*Number of individuals*	48	52	
**No ProF use**, n (%)			
Findability	20 (42)	12 (23)	0.055
Time	16 (33)	15 (29)	0.628
Effort	5 (10)	1 (2)	0.102
Importance	13 (27)	1 (2)	0.000
Grade	21 (44)	20 (38)	0.591
Utility	0 (0)	6 (12)	0.027^d^
Answer key	1 (2)	5 (10)	0.207^d^
Other	3 (6)	10 (19)	0.054

ProF, progress test feedback system

^a^ Chi-squared test

^b^ Subgroup analysis; Perceived formative/summative: students in the formative/summative test group who knew it was formative/summative

^c^ Findability: “I do not know where I can find the feedback”; Time: “I did not have time to look at the feedback”;

Effort: “I did not put effort in this progress test”; Importance: “I thought this progress test was not important”;

Grade: “I got a pass/good for this progress test”; Utility: “I find the feedback not useful”;

Answer key: “I already checked my answers with the answer key”

^d^ Fisher’s exact test

Qualitative data revealed that **representation** of the *formative* test also played a role in feedback consultation (Fig. 2). Performance-oriented students indicated less interest in the feedback of the *formative* PT, because their low test-taking effort in taking the *formative* PT did not provide a valid representation of their own knowledge level. Therefore, the feedback was less meaningful to them:

"I think I took a quick look at ProF. That I just looked at that line, but that I thought yes, it is probably now higher than it should be. So I did not attach much value to it.” (Interview #4).

This was also the case for students who used study material during the *formative* PT. Besides, these students found it more useful to receive direct feedback during the PT. In contrast, for learning-oriented students who used the *formative* PT as self-assessment the test result was a valid representation, and they were interested to consult the feedback to assess their strengths and weaknesses.

ProF consultation was relatively high after the first *formative* PT (September 2020, Online Resource 4). The interviewed students mentioned that this could be explained by curiosity right after switching to formative testing (Fig. 2, ‘***Switch***’):

"The first time it is always exciting, oh new and what would be my result now that it’s online for the first time. And is there a difference maybe with the paper version that I always had. So then it is a bit more interesting and if you’ve done a few then you just think oh it’s going fine, whatever.” (Interview #18).

### Active feedback use

The internal consistency (Cronbach’s α) of the 6-point Likert scale items was 0.85 (> 0.80: acceptable) (Lance et al., 2006; Nunnaly, 1994). After deletion of item 7 of the subfactor ENJ the Cronbach’s α remained 0.85. We found no difference in the mean total score on the items of AUF and ENJ (3.2 (0.9) versus 3.1 (0.9), t(223): 1.09, 95% CI -0.10-0.36). Comparing perceived assessment conditions yielded the same result (Table 2). On item level, item 7 (enjoy) had the highest score (5 (4–6), whereas item 3 (setting goals) and 6 (changing learning) had the lowest score (2 (1–3) in both groups (Online Resource 11).

Students who were interested in the test result and feedback often only consulted the feedback without actively using it. They seemed to use the feedback as a ’thermometer‘ to assess if they were still at the right ’temperature‘. If they were still on the right track, they did not feel the urgency to change anything and engage with the feedback: “*If it ain’t broke, don’t fix it*”*(# Interview 4*). An insufficient grade on the other hand was or will be an incentive to act on the feedback and use it to prepare for the next PT:

"Suppose, if I had failed I would think oh dear, then I will really look at what I did wrong, which subject and really do that because you still want to get those study credits.” (Interview #18).

Although the *formative* PT was also graded, grade focus only occurred in the *summative* PT, mainly because an insufficient grade on the *formative* PT had no consequences. Thus, no need was felt to act on the feedback (Fig. 2).

## Discussion

In this mixed-methods study, we compared the effect of a purely formative PT (*formative* PT) with a PT with a summative component (*summative* PT) on medical students’ feedback use and test-taking motivation. We triangulated quantitative and qualitative interview data to explain these in the context of a *formative* versus a *summative* PT. Our thematic map (Fig. 2), based on our qualitative data, in which EVT and goal-orientation frameworks were integrated helped explain our quantitative results, and provided a nuanced picture of the different ways students approached the feedback in the *formative* and *summative* PT. Test preparation was relatively low for both PT assessment conditions and did not differ between groups. Qualitative data showed that test-taking motivation and feedback use relate to how students value the assessment. Performance-oriented students valued the *summative* PT as more important because of its’ consequences for study progress, and learning-oriented students valued the PT feedback for their own learning, regardless of the assessment condition. These orientations influenced their test-taking behaviour (effort and strategy), and feedback consultation (representation of *formative* PT results). Self-reported questionnaire data showed more ProF consultation and use of the answer key after the *summative* PT compared to the *formative* PT. Actual feedback use, measured by ProF logging data, showed the same results. Students in the *formative* PT-group who did not consult PT feedback more often reported the *formative* PT as unimportant, reflecting the perceptions of performance-oriented students. However, t self-reported active feedback use after the PT was relatively low in general and did not differ between groups, which was mainly determined by grade focus.

### Test preparation, feedback consultation and test-taking motivation

We measured test preparation and feedback consultation with different data sources. The ProF logging data demonstrate that, in general, students made limited use of ProF to consult feedback, which is important to take into account with the interpretation of our data. Despite low use of ProF, our data can contribute to a better understanding of feedback behaviour. Both questionnaire and interview results suggest that the motivation to prepare and consult feedback relates to how students value the assessment. The interview results revealed that experienced utility value (i.e. usefulness) and attainment value (i.e. importance) of the PT affected test-taking effort, the important component of test-taking motivation, which influenced feedback consultation (Eccles & Wigfield, 2020). This positive relation between value and effort has also been found in test-taking motivation with test performance as outcome (Cole et al., 2008; Penk & Schipolowski, 2015; Zilberberg et al., 2014).

Moreover, our interview data showed that students valued the different PT assessment conditions based on whether they were orientated towards performance or learning. This aligns with the goal-orientation theory, which states that performance-orientated students focus on achievement based on normative standards (i.e. study credits), whereas learning-orientated students focus on achievement based on learning (Elliot & Dweck, 2005). It seems that students’ goal orientation guided their test effort and engagement with the feedback. Although students did not explicitly state goals for the PT in our study, they did show a more general focus on either learning or performance. Below, we elaborate on students’ performance and learning orientation in this study.

### Performance-oriented students

Performance-orientated feedback behaviour was revealed by our qualitative interview data, and confirmed by our quantitative results. The interviews showed that students found the *summative* PT more important and valuable, which led to less test-taking effort and feedback consultation after the *formative* PT. Quantitative data confirmed this performance orientation as self-reported, and actual feedback consultation was higher after the *summative* PT. Also, the perception that the PT was not important, and thus ProF consultation or preparation was not needed, was more profound in students who participated in the *formative* PT. These results are visualized in the upper path of our thematic map (Fig. 2). The performance-oriented students mainly focus on the direct personal consequences (i.e., study-credits) of the test in the pre-assessment phase, see no value in investing effort in the *formative* tests (i.e., low effort during assessment), leading to an invalid representation of their test result, and a decreased motivation to consult the feedback of the *formative* test post-assessment. The study credits in the *summative* test, on the other hand, motivated these students to consult the feedback.

### Learning-oriented students

Our qualitative analysis suggested that students were not also focused on learning. Thus, the interview data further deepened and nuanced our understanding of students’ feedback use. Learning-orientated students valued the PT and its feedback as part of their learning process, regardless of the assessment condition (Fig. 2, lower path). These students took the *formative* test seriously, invested high effort, used it for self-assessment or self-study, and were motivated to consult the feedback of the *formative* test. This aligns with previous research in surgical residents showing that formative assessments promoted a learning-oriented motivation (Lund et al., 2022). The strategy of self-study was interesting in that the test itself was used as tool to pay attention to knowledge gaps and generate direct feedback. It is more likely that these students benefit from formative assessments and engage in more self-regulated learning compared to students adopting the performance orientation (Ames, 1992; Pintrich, 2000; Schunk et al., 2008).

Some learning-oriented students indirectly also focused on the study credits considering that the *formative* PTs would switch back to *summative*. Figure 2 shows that this contextual factor (‘*Temporariness’*) influenced test-taking effort, and strategy in the *formative* test of these students (with the dotted arrows). They decided to put high effort in the *formative* test, and use it as self-assessment, to make sure they were at the right level to pass the upcoming *summative* test. Although these students predominantly focus on learning in the *formative* PTs, they do not completely let go their performance-orientation for the study credits of the future *summative* PTs.

### Active use of feedback

Besides students’ feedback consultation in the e-mail, ProF or by using the answer key, which can be considered a more passive use of feedback, we also measured active use of feedback after the PT by the AUF scale items in our questionnaire (Brown et al., 2016). Although students enjoyed receiving feedback, active use of feedback after the PT was relatively low and no difference was found between the groups. The interview data also showed that most students did not actively use the feedback, and that they tended to act only on the feedback when they failed on the *summative* PT. This is illustrated in Fig. 2 in the post-assessment phase, where all students, regardless of their orientation, were driven by the grade in their decision to act on the feedback after consultation. This suggests that failure drove using feedback, regardless of students’ learning orientation. However, we could only find qualitative evidence for this, as too few students failed the PT to provide quantitative evidence. As described in previous literature, grade focus strongly limits the likelihood to engage with feedback after a sufficient summative grade (Harrison et al., 2015; Winstone et al., 2017). However, students stated even less engagement with the feedback after the *formative* PT, because they lacked a feeling of urgency to change something as this test had no direct consequences for their study progress. These findings are in line with earlier studies on progress testing, where the effect of the feedback on learning was questionable (Aarts et al., 2010; Given et al., 2016; Schüttpelz-Brauns et al., 2018; Van Der Vleuten et al., 1996; Yielder et al., 2017). Although students used the feedback to monitor their progress, and identify strengths and weaknesses in these studies, there was no direct influence on future learning (Aarts et al., 2010; Given et al., 2016).

### Implications for practice

Our results suggest that the desired positive effect of formative testing on the learning process is limited in progress testing, with students mainly focusing on performance. Introducing more formative assessments in medical education requires a change in shift in focus towards the learning process (learning-oriented) rather than the outcome (performance-oriented), and enhancing students’ feedback literacy: their ability to effectively engage with and utilize feedback (Molloy et al., 2020). This involves creating a supportive environment in which students are encouraged to develop feedback literate skills (Carless & Winstone, 2020). An example of such a system emphasizing the value of assessment for learning is the programmatic assessment approach. In this approach assessments are no longer divided in formative and summative, but rather represent a continuum of stakes (from low to high). Heeneman et al. demonstrated positive results on feedback use of embedding a formative PT in a programmatic assessment system, in which the reflection on the PT, and guidance in the feedback process by mentors in the curriculum is embedded (Heeneman et al., 2017). A supportive assessment environment that emphasizes the understanding of the concept, and purpose of formative testing is key in motivating students and support learning (Heeneman et al., 2015; Nouns & Georg, 2010).

### Strengths and limitations

In the present study we had the unique opportunity to make a direct comparison between two conditions of the same test in one medical curriculum. Except for the assessment conditions, the educational setting was exactly the same for all students and feedback was provided to all students, which facilitated the assessment of the (additional) effect of the summative component over the formative component of assessment on feedback use. Moreover, we analysed both assigned and perceived test conditions, which showed the same trend. Additionally, triangulation of quantitative, and qualitative data was used to increase validity and create a more in-depth understanding of student’s values. The triangulation of three data sources also adds to the credibility of our conclusion that the formative PT was associated with less feedback use than the summative PT.

This study also has some limitations. Firstly, this study was conducted at only one medical school, which could limit the transferability to other settings. Secondly, we cannot completely rule out that the difference in study-years between the groups affected feedback behaviour. As third-year students are more experienced with the PT, possibly having a more serious attitude towards their study, this might have resulted in a higher baseline level of feedback use. Nevertheless, test preparation was similar between groups, and the effect found in the ProF logging data remained significant after adjusting for previous ProF use in both years. Moreover, the interview data clarified that the formative and summative component of the PT played a significant role in their feedback behaviour, regardless of their study progress. Thirdly, the responders to our questionnaires were overall students with more ProF logging sessions, and the response rate of the students in the formative PT-group was relatively low. However, the ProF logging data of all students, both responders and non-responders, point towards the same conclusion that feedback consultation was higher after the *summative* PT. Fourthly, the assessment of a more longitudinal pattern of ProF logging behaviour under summative conditions was hindered by changes in PT conditions before September 2021 (COVID-19) and after February 2022 (new adaptive format). Finally, it must be noted that this study focused on the PT, which is a longitudinal, repetitive and comprehensive assessment. We cannot be sure to what extent these results can be adapted to other contexts, such as a context with a different assessment structure or to end-of-course examinations. The perception of feedback and the feedback behaviour in these other contexts is an interesting question for future research. Moreover, additional research is needed to understand the interaction between the different goal orientations and feedback use.

## Conclusion

In conclusion, this study found that students make little use of PT feedback. When they do use PT feedback, a *summative* PT is associated with more feedback consultation compared to a *formative* PT, which can be explained by lower overall test-taking motivation in the *formative* PT and a performance-orientation. Nonetheless, qualitative data also showed learning-oriented students who found the *formative* PT useful and important for their learning, emphasizing that the perceived value of assessment is key to the learning effect of formative testing. Active use of feedback after the PT was low in both assessment conditions and seemed to be affected mostly by high-stakes consequences (i.e., not obtaining enough study credits due to failing the *summative* PT). This might be partly because reflection, and guidance in the feedback process were not embedded in the curriculum. Therefore, it is important to consider the introduction of formative assessments in the medical curriculum very carefully, and make sure students understand its value and are supported in the feedback process.

## Electronic supplementary material

Below is the link to the electronic supplementary material.

Supplementary Material 2

Supplementary Material 3

Supplementary Material 4

Supplementary Material 5

Supplementary Material 6

Supplementary Material 7

Supplementary Material 8

Supplementary Material 9

Supplementary Material 10

Supplementary Material 11

Supplementary Material 1
