# Supplementary material for: Does ‘summative’ count? The influence of the awarding of study credits on feedback use and test-taking motivation in medical progress testing
Source: Adv Health Sci Educ Theory Pract. 2024 Mar 19;29(5):1665–88. doi: 10.1007/s10459-024-10324-4 (PMC11549188; doi:10.1007/s10459-024-10324-4)
Supplement: Supplementary file 1 — Supplementary Material 2 [file 10459_2024_10324_MOESM2_ESM.pdf]

Does 'summative' count? The influence of the awarding of study credits on feedback use and test-taking behaviour in medical progress testing

Elise V. van Wijk, Floris M. van Blankenstein, Jeroen Donkers, Roemer J. Janse, Jacqueline Bustraan, Liesbeth G.M. Adelmeijer, Eline A. Dubois, Friedo W. Dekker, Alexandra M.J. Langers*

***Corresponding author**:

Department of Gastroenterology and Hepatology, Leiden University Medical Center, the Netherlands

Leiden University Medical Center, Albinusdreef 2, 2333 ZA, Leiden, The Netherlands

Email: *a.m.j.langers@lumc.nl*

Journal: Advances in Health Sciences Education

Online Resource 2. Questionnaire Progress Test and Feedback Behaviour

In the context of the study on the effect of different assessment conditions of the progress test on learning behaviour we would like to conduct a short questionnaire about the **last progress test on the 2nd of February 2022.**

Your student number will only be used to link the questionnaire data with data from ProF (logging data and PT results) and uSis (gender, age and study-year). After linking the data, your data will be encrypted by an independent person. This person is not directly involved in this study and will be the only person who has access to the decryption key. The principal investigator will only have access to the coded data which will be used for the statistical analyses. The results of these analyses will be reported on group level and will therefore not be traceable to individual persons.

Informed Consent

- I have read the information letter. I was given the opportunity to ask additional questions and I have received a clear answer to my questions. I have been given enough time to decide whether I wanted to participate.
- I know that my participation is completely voluntary and that I can withdraw my consent to participate at any time without giving a reason.
- I give permission for the collection, storage and use of my data for the above mentioned purposes of the research.
- I know that only the principal investigator has access to my personal data. I know that only the principal investigator and her supervisor can view the anonymized data. The names of the principal investigator and her supervisor are listed in the information letter.
- I give permission to keep my data for another fifteen years after the end of this research for possible further analysis.

O I agree to the use of my (anonymized) answers to the questionnaire for the purpose of this research.

This questionnaire only concerns the **progress test on the 2nd of February 2022.**
ProF refers to the online feedback system of the progress test.

For part of the students the result of this progress test did not count towards the awarding of credits, while for another part of the students it did. Indicate what applies to you.

1. Did the result of this progress test count towards the awarding of credits?
   - Yes
   - No
   - Don't know

2. How important was this progress test for you (e.g. for obtaining credits, for your study progress, personal reasons). *Choose one answer option.*
   - Low
   - Intermediate
   - High

The following questions relate to the preparation prior to the progress test on the 2nd of February 2022.

3. Did you prepare for this progress test? *Choose one answer option.*
   - Yes
   - No

*In case you answered **question 3** with **"yes"**, you can continue with question 5 and skip question 4. In case you answered **question 3** with **"no"**, continue with question 4.*

4. Why did you not prepare for this VGT? *Multiple answers possible.*
   - I had no time to prepare
   - I did not feel like preparing
   - I always pass my progress test without preparation
   - I got a pass/good for my previous progress test
   - I thought this progress test was not important
   - Other: *<free text>*

The following questions relate to the consultation of the feedback after the progress test on the 2nd of February 2022.

5. Did you check the answers of this progress test with the answer key?
   - Yes
   - No

6. Did you look at the feedback of this progress test in the email?
   - Yes
   - No

7. Did you consult ProF to look at the feedback of this progress test?
   - Yes
   - No

*In case your answer to **question 9 was "no"**, you can continue with question 11 and skip question 10. In case you answered **"yes" to question 9**, you can continue with question 10 and skip question 11.*

8. What is the reason that you did not look at the feedback in ProF? *Multiple answers possible.*
   - I do not know where I can find the feedback

- I did not have time to look at the feedback
- I did not put effort in this progress test
- I thought this progress test was not important
- I had a pass/good for this progress test
- I find the feedback not useful
- I already checked my answers with the answer key
- Other: *<free text>*

9. Which section of ProF did you look at? *Multiple answers possible.*
   - Progress total score (longitudinal)
   - Total score of this progress test (moment)
   - Progress on discipline score (longitudinal)
   - Discipline score of this progress test (moment)
   - Progress on category score (longitudinal)
   - Category score of this progress test (moment)
   - Progress on cluster score (longitudinal)
   - Cluster score of this progress test (moment)
   - I do not know

The following seven items relate to the <u>consultation of feedback in the email or ProF after</u> the progress test on the 2nd of February 2022. For the statements below, indicate the extent to which you agree or disagree with each statement.

Totally disagree(1) Mostly disagree(2) Slightly agree(3) Moderately agree(4) Mostly agree(5) Strongly agree(6)

1. **I actively use the feedback to help me improve.**
2. **I pay attention to the feedback.**
3. **I use the feedback to set goals for the next progress test.**
4. **I look at the feedback to see what I did wrong.**
5. **The feedback makes me try harder.**
6. **The feedback changes the way I learn and study.**
7. **I enjoy getting the feedback.**
