# Supplementary material for: Does ‘summative’ count? The influence of the awarding of study credits on feedback use and test-taking motivation in medical progress testing
Source: Adv Health Sci Educ Theory Pract. 2024 Mar 19;29(5):1665–88. doi: 10.1007/s10459-024-10324-4 (PMC11549188; doi:10.1007/s10459-024-10324-4)
Supplement: Supplementary file 2 — Supplementary Material 3 [file 10459_2024_10324_MOESM3_ESM.pdf]

Does 'summative' count? The influence of the awarding of study credits on feedback use and test-taking behaviour in medical progress testing

Elise V. van Wijk, Floris M. van Blankenstein, Jeroen Donkers, Roemer J. Janse, Jacqueline Bustraan, Liesbeth G.M. Adelmeijer, Eline A. Dubois, Friedo W. Dekker, Alexandra M.J. Langers*

***Corresponding author**:
Department of Gastroenterology and Hepatology, Leiden University Medical Center, the Netherlands
Leiden University Medical Center, Albinusdreef 2, 2333 ZA, Leiden, The Netherlands
Email: *a.m.j.langers@lumc.nl*

Journal: Advances in Health Sciences Education

Online Resource 3. Questions of semi-structed individual interviews

Part 1. Own feedback experiences

1.Do you prepare for the progress test?
How do you prepare?
What determines whether you prepare for the progress test?

2. Do you consult the result of the progress test?
Which methods do you use to consult the test result?
What determines whether you look at the test result? And what is the role of the test condition in this?

3. Do you use the result of the progress test?
What do you do with this information?
What determines whether you use the feedback? And what is the role of the test condition in this?

4. Are you aware of the online feedback system (ProF)? *Only asked if students did not mention ProF yet.*
Why are you not using this feedback system?
What do you think is the reason that you are not aware of ProF?

Part 2. Reflection and interpretation of ProF logging data (graph)

1.Can you describe what you see?

2.What do you think when you see these data?

3. How would you explain and/or interpret these data?

Part 3. Perception of progress test and feedback

1.What is your perception of the progress test? And which place does it have in your study program?

2.What is your perception of the way(s) the test result is presented to you?
Do you have any suggestions to improve this?
