# Supplementary material for: Does ‘summative’ count? The influence of the awarding of study credits on feedback use and test-taking motivation in medical progress testing
Source: Adv Health Sci Educ Theory Pract. 2024 Mar 19;29(5):1665–88. doi: 10.1007/s10459-024-10324-4 (PMC11549188; doi:10.1007/s10459-024-10324-4)
Supplement: Supplementary file 4 — Supplementary Material 5 [file 10459_2024_10324_MOESM5_ESM.pdf]

Does 'summative' count? The influence of the awarding of study credits on feedback use and test-taking behaviour in medical progress testing

Elise V. van Wijk, Floris M. van Blankenstein, Jeroen Donkers, Roemer J. Janse, Jacqueline Bustraan, Liesbeth G.M. Adelmeijer, Eline A. Dubois, Friedo W. Dekker, Alexandra M.J. Langers*

***Corresponding author**:
Department of Gastroenterology and Hepatology, Leiden University Medical Center, the Netherlands
Leiden University Medical Center, Albinusdreef 2, 2333 ZA, Leiden, The Netherlands
Email: *a.m.j.langers@lumc.nl*

Journal: Advances in Health Sciences Education

Online Resource 5. Descriptive characteristics of interviewees

**Number of logging sessions**[a]	**0**		**1**		**2-4**		**>5**		
**Grade**	Fail	Pass/Good	Fail	Pass/Good	Fail	Pass/Good	Fail	Pass/Good	**Total year (M/F)**
Year 2	11[c]	3[c], 10[c]		21[c]		20[b],19[c]			6 (1/5)
Year 3	7[c]	18[c]	4[b]	9[c]		5[b], 8[b]	2[c]	15[b]	8 (4/4)
Year 5	6[c]		14[c]		16[c]	17[b]			4 (1/3)
Year 6	13[b]			1[c]		12[b]			3 (2/1)
**Total Fail and Pass/Good (M/F)**	4 (1/3)	3 (0/3)	2 (1/1)	3 (0/3)	1 (0/1)	6 (5/1)	1 (0/1)	1 (1/0)	

[a] ProF logging sessions from September 2020 to January 2021. M = male, F = female.
[b] Male. [c] Female.
