# Supplementary material for: Does ‘summative’ count? The influence of the awarding of study credits on feedback use and test-taking motivation in medical progress testing
Source: Adv Health Sci Educ Theory Pract. 2024 Mar 19;29(5):1665–88. doi: 10.1007/s10459-024-10324-4 (PMC11549188; doi:10.1007/s10459-024-10324-4)
Supplement: Supplementary file 6 — Supplementary Material 7 [file 10459_2024_10324_MOESM7_ESM.pdf]

Does 'summative' count? The influence of the awarding of study credits on feedback use and test-taking behaviour in medical progress testing

Elise V. van Wijk, Floris M. van Blankenstein, Jeroen Donkers, Roemer J. Janse, Jacqueline Bustraan, Liesbeth G.M. Adelmeijer, Eline A. Dubois, Friedo W. Dekker, Alexandra M.J. Langers*

***Corresponding author**:
Department of Gastroenterology and Hepatology, Leiden University Medical Center, the Netherlands
Leiden University Medical Center, Albinusdreef 2, 2333 ZA, Leiden, The Netherlands
Email: *a.m.j.langers@lumc.nl*

Journal: Advances in Health Sciences Education

Online Resource 7. Baseline characteristics of responders and non-responders of the questionnaire.

	**Responders** (*n* = 264)	**Non-responders** (*n* = 354)	**SMD**[a]
Age, median (IQR)	21 (20, 21)	21 (20, 22)	0.107
Female, n (%)	185 (70)	244 (69)	0.022
Grade, n (%)			
Fail	24 (9)	64 (18)	0.266
Pass	106 (40)	196 (55)	0.304
Good	134 (51)	94 (27)	0.508
Proportion passed earlier PTs, %			
Sep '21	223 (87)	286 (85)	0.058
Dec '21	226 (87)	249 (73)	0.355
ProF sessions, mean (SD)[b]	1.29 (1.60)	0.71 (1.87)	0.334

SMD, standardized mean difference. IQR, interquartile range. PT: progress test.
[a] A standardized mean difference >0.1 may point towards meaningful imbalance between groups.
[b] Period of consultation for each PT starts the week before the PT and ends the week before the next PT.
