# Supplementary material for: Does ‘summative’ count? The influence of the awarding of study credits on feedback use and test-taking motivation in medical progress testing
Source: Adv Health Sci Educ Theory Pract. 2024 Mar 19;29(5):1665–88. doi: 10.1007/s10459-024-10324-4 (PMC11549188; doi:10.1007/s10459-024-10324-4)
Supplement: Supplementary file 7 — Supplementary Material 8 [file 10459_2024_10324_MOESM8_ESM.pdf]

Does 'summative' count? The influence of the awarding of study credits on feedback use and test-taking behaviour in medical progress testing

Elise V. van Wijk, Floris M. van Blankenstein, Jeroen Donkers, Roemer J. Janse, Jacqueline Bustraan, Liesbeth G.M. Adelmeijer, Eline A. Dubois, Friedo W. Dekker, Alexandra M.J. Langers*

***Corresponding author**:
Department of Gastroenterology and Hepatology, Leiden University Medical Center, the Netherlands
Leiden University Medical Center, Albinusdreef 2, 2333 ZA, Leiden, The Netherlands
Email: *a.m.j.langers@lumc.nl*

Journal: Advances in Health Sciences Education

Online Resource 8. Students' idea of assessment condition and perceived stakes of the progress test.

	**Formative Test** (*n* = 113)	**Summative Test** (*n* = 151)
**Students' idea of condition (Q1)**, n (%)		
Formative	79 (70)	8 (5)
Summative	11 (10)	128 (85)
Don't know	23 (20)	14 (9)
**Perceived stakes (Q2)**, n (%)		
Low	57 (50)	19 (13)
Intermediate	48 (42)	94 (62)
High	8 (7)	37 (25)

Q1: Did the result of this progress test count towards the awarding of credits?
Q2: How important was this progress test for you (e.g. for receiving credits, for your study progress, personal reasons)?
