# Supplementary material for: Does ‘summative’ count? The influence of the awarding of study credits on feedback use and test-taking motivation in medical progress testing
Source: Adv Health Sci Educ Theory Pract. 2024 Mar 19;29(5):1665–88. doi: 10.1007/s10459-024-10324-4 (PMC11549188; doi:10.1007/s10459-024-10324-4)
Supplement: Supplementary file 8 — Supplementary Material 9 [file 10459_2024_10324_MOESM9_ESM.pdf]

Does ‘summative’ count? The influence of the awarding of study credits on feedback use and test-taking behaviour in medical progress testing

Elise V. van Wijk, Floris M. van Blankenstein, Jeroen Donkers, Roemer J. Janse, Jacqueline Bustraan, Liesbeth G.M. Adelmeijer, Eline A. Dubois, Friedo W. Dekker, Alexandra M.J. Langers*

***Corresponding author**:
Department of Gastroenterology and Hepatology, Leiden University Medical Center, the Netherlands
Leiden University Medical Center, Albinusdreef 2, 2333 ZA, Leiden, The Netherlands
Email: *a.m.j.langers@lumc.nl*

Journal: Advances in Health Sciences Education

Online Resource 9. Reasons for not preparing the *formative* or *summative* progress test.

	Formative Test	Summative Test	p-value[a]
**True formative and summative**			
Number of individuals	99	123	
**No preparation**, n (%)			
Time[c]	35 (35)	42 (34)	0.765
Motivation	18 (18)	11 (9)	0.036
Need	69 (70)	92 (75)	0.543
Grade	39 (39)	46 (37)	0.671
Importance	27 (27)	1 (1)	0.000
Other	5 (5)	6 (5)	1.000[b]
**Perceived formative and summative[b]**			
Number of individuals	71	103	
**No preparation**, n (%)			
Time	27 (38)	35 (34)	0.491
Motivation	14 (20)	10 (10)	0.050
Need	45 (63)	78 (76)	0.134
Grade	29 (41)	39 (38)	0.584
Importance	26 (37)	0 (0)	0.000
Other	4 (6)	4 (4)	0.715[b]

[a] Chi-squared test.
[b] Subgroup analysis; Perceived formative: students in the formative test group who knew it was formative; Perceived summative: students in the summative test group who knew it was summative.
[c] Time: “I had no time to prepare”;
Motivation: “I did not feel like preparing”;
Need: “I always pass my progress test without preparation”;
Grade: “I got a pass/good for my previous progress test”;
Importance: “I thought this progress test was not important”.
[b] Fisher’s exact test.
