# Supplementary material for: Does ‘summative’ count? The influence of the awarding of study credits on feedback use and test-taking motivation in medical progress testing
Source: Adv Health Sci Educ Theory Pract. 2024 Mar 19;29(5):1665–88. doi: 10.1007/s10459-024-10324-4 (PMC11549188; doi:10.1007/s10459-024-10324-4)
Supplement: Supplementary file 9 — Supplementary Material 10 [file 10459_2024_10324_MOESM10_ESM.pdf]

Does 'summative' count? The influence of the awarding of study credits on feedback use and test-taking behaviour in medical progress testing

Elise V. van Wijk, Floris M. van Blankenstein, Jeroen Donkers, Roemer J. Janse, Jacqueline Bustraan, Liesbeth G.M. Adelmeijer, Eline A. Dubois, Friedo W. Dekker, Alexandra M.J. Langers*

***Corresponding author**:
Department of Gastroenterology and Hepatology, Leiden University Medical Center, the Netherlands
Leiden University Medical Center, Albinusdreef 2, 2333 ZA, Leiden, The Netherlands
Email: *a.m.j.langers@lumc.nl*

Journal: Advances in Health Sciences Education

Online Resource 10. Average ProF logging sessions for each progress test

		N[a]	Average ProF sessions, mean (95% CI)	p-value[b]
PT sep '21[c,d]	Year 2	316	0.67 (0.52-0.82)	
	Year 3	305	1.25 (1.03-1.46)	
PT dec '21	Year 2	316	0.38 (0.19-0.57)	
	Year 3	305	0.61 (0.44-0.79)	
PT feb '22	Year 2	316	0.39 (0.27-0.51)	
	Year 3	305	0.83 (0.66-1.01)	<0.001

ProF, progress test feedback system; PT, progress test.
[a] Population based on participants of the PT in February.
[b] Unpaired t-test.
[c] PT feb '22 summative for year 3, formative for year 2.
[d] Period of consultation for each PT starts the week before the PT and ends the week before the next PT.
