# Supplementary material for: Does ‘summative’ count? The influence of the awarding of study credits on feedback use and test-taking motivation in medical progress testing
Source: Adv Health Sci Educ Theory Pract. 2024 Mar 19;29(5):1665–88. doi: 10.1007/s10459-024-10324-4 (PMC11549188; doi:10.1007/s10459-024-10324-4)
Supplement: Supplementary file 10 — Supplementary Material 11 [file 10459_2024_10324_MOESM11_ESM.pdf]

**Does ‘summative’ count? The influence of the awarding of study credits on feedback use and test-taking behaviour in medical progress testing**

Elise V. van Wijk, Floris M. van Blankenstein, Jeroen Donkers, Roemer J. Janse, Jacqueline Bustraan, Liesbeth G.M. Adelmeijer, Eline A. Dubois, Friedo W. Dekker, Alexandra M.J. Langers*

***Corresponding author**:
Department of Gastroenterology and Hepatology, Leiden University Medical Center, the Netherlands
Leiden University Medical Center, Albinusdreef 2, 2333 ZA, Leiden, The Netherlands
Email: *a.m.j.langers@lumc.nl*

**Journal**: Advances in Health Sciences Education

**Online Resource 11.** Median scores (IQR) of the 6-point Likert scale items of “*Active use of feedback*” and its subfactor “*Enjoyment*” in the *formative* and *summative* progress test-group.

| | **Formative Test** | **Summative Test** |
|---|---|---|
| **True formative and summative** | | |
| Number of individuals[a] | 91 | 135 |
| **Feedback use,** median (IQR) | | |
| Item 1[c] | 3 (2-3) | 3 (2-3) |
| Item 2 | 3 (3-4) | 3 (2-4) |
| Item 3 | 2 (1-3) | 2 (1-3) |
| Item 4 | 4 (3-5) | 4 (3-5) |
| Item 5 | 3 (2-4) | 3 (2-4) |
| Item 6 | 2 (1-3) | 2 (1-3) |
| Item 7 | 5 (4-6) | 5 (4-6) |
| **Perceived formative and summative[b]** | | |
| Number of individuals | 62 | 114 |
| **Feedback use,** median (IQR) | | |
| Item 1 | 3 (2-3) | 3 (2-3) |
| Item 2 | 3 (3-4) | 3 (2-4) |
| Item 3 | 2 (1-3) | 2 (3-4) |
| Item 4 | 4 (3-5) | 4 (3-4) |
| Item 5 | 3 (2-4) | 3 (2-4) |
| Item 6 | 2 (1-3) | 2 (1-3) |
| Item 7 | 5 (4-6) | 5 (4-6) |

IQR, interquartile range.
[a] Students who consulted feedback in e-mail or progress test feedback system.
[b] Subgroup analysis; Perceived formative: students in the formative test group who knew it was formative; Perceived summative: students in the summative test group who knew it was summative.
[c] Item 1 = I actively use the feedback to help me improve;
Item 2 = I pay attention to the feedback;
Item 3 = I use the feedback to set goals for the next progress test;
Item 4 = I look at the feedback to see what I did wrong;
Item 5 = The feedback makes me try harder;
Item 6 = The feedback changes the way I learn and study;
Item 7 = I enjoy getting the feedback.
