# Supplementary material for: Does ‘summative’ count? The influence of the awarding of study credits on feedback use and test-taking motivation in medical progress testing
Source: Adv Health Sci Educ Theory Pract. 2024 Mar 19;29(5):1665–88. doi: 10.1007/s10459-024-10324-4 (PMC11549188; doi:10.1007/s10459-024-10324-4)
Supplement: Supplementary file 11 — Supplementary Material 1 [file 10459_2024_10324_MOESM12_ESM.pdf]

Does ‘summative’ count? The influence of the awarding of study credits on feedback use and test-taking behaviour in medical progress testing

Elise V. van Wijk, Floris M. van Blankenstein, Jeroen Donkers, Roemer J. Janse, Jacqueline Bustraan, Liesbeth G.M. Adelmeijer, Eline A. Dubois, Friedo W. Dekker, Alexandra M.J. Langers*

***Corresponding author**:

Department of Gastroenterology and Hepatology, Leiden University Medical Center, the Netherlands Leiden University Medical Center, Albinusdreef 2, 2333 ZA, Leiden, The Netherlands

Email: *a.m.j.langers@lumc.nl*

Journal: Advances in Health Sciences Education

Online Resource 1. Example of feedback of the progress test with the results per category and per discipline provided by e-mail

		Individual				Test moment group (n=57)							
**Description categories**	**Number of questions**	**Correct**	**Incorrect**	**?**	**Score**	**Correct**	**Std**	**Incorrect**	**Std**	**?**	**Std**	**Score**	**Std**
01 Respiratory system	13	69	31	0	56	68	13	28	12	4	7	57	18
02 Musculoskeletal system	17	59	41++	0--	38	58	11	31	9	11	10	44	14
03 Mental Health Care	16	69	31+	0	-58	75	14	20	12	5	7	68	18
04 Reproductive system	11	-45	55++	0	27--	58	15	29	13	13	13	48	18
05 Blood, lymph, heart and circulation	24	58	25	17+	48	60	13	29	11	11	9	48	17
06 Hormones and metabolism	13	-46	46++	8	-29	57	13	31	14	12	10	46	17
07 Skin and connective tissue	12	83	17	0	78	80	10	17	10	3	6	74	13
08 Personal, social and prevention aspects	17	29--	71++	0--	-4--	52	14	38	14	11	10	35	19
09 Digestive system	17	71	29	0--	61	66	12	26	11	8	7	57	15
10 Kidneys and urinary tract	16	69	25	6	59	71	13	21	11	7	8	63	16
11 Nervous system and senses	17	47--	47++	-6	28--	62	13	26	12	12	11	53	16
12 Knowledge about skills	23	48	39	13	33	49	11	40	11	11	9	32	14
**Total**	196	-57	38++	-5	-42	62	8	29	6	9	6	51	9

-/--/++/+ low respectively high in comparison with the total group. Results are presented in percentages. Std = standard deviation. ? = question mark option use.

		Individual				Test moment group (n=57)							
**Description disciplines**	**Number of questions**	**Correct**	**Incorrect**	**?**	**Score**	**Correct**	**Std**	**Incorrect**	**Std**	**?**	**Std**	**Score**	**Std**
Anatomy	12	58	33	8	46	60	15	34	14	6	9	48	20
Biochemistry, molecular and cellular biology and genetics	18	50	44++	6--	34	46	14	31	12	24	14	34	17
Pharmacology	8	62	25	12	54	65	15	27	14	8	9	54	20
Physiology	11	73	27+	0-	62	73	17	18	12	9	12	65	21
Patho-, immuno- en microbiology	10	50	40	10	33-	57	15	34	15	10	10	44	19
**Basic-, supportive subjects**	59	58	36+	7-	44	58	9	29	7	13	8	47	10
Epidemiology/statistics	7	71+	29	0-	57+	55	23	32	15	12	21	41	26
Metamedica	5	20--	80++	0-	-23--	51	23	38	23	11	14	32	33
Psychiatry/psychology	12	67	33++	0-	54-	73	14	20	12	7	10	65	17
Social medicine	3	33	67+	0-	0	42	26	51	28	8	15	17	37
**Behavioural scientific/other subjects**	27	56	44++	0-	35-	61	13	30	9	9	10	47	15
Surgery	16	69	31	0-	56	67	13	27	12	6	8	56	17
Dermatology/ENT/opthalmology	14	57	36	7	44	63	14	29	14	8	10	53	18
Geriatrics	8	62	38+	0	44	68	17	29	16	3	6	55	23
Obstetrics/Gynaecology	7	43--	57++	0-	21--	60	14	28	17	13	14	49	19
Family medicine	20	40--	55++	5	21--	61	12	34	12	4	5	49	16
Internal medicine	26	73	19	8+	67	73	11	22	9	5	5	64	14
Paediatrics	12	50-	42++	8	32-	60	15	28	13	12	12	48	19
Neurology	7	43	43+	14	19-	50	17	32	17	18	19	37	21
**Clinical subjects**	110	57-	37++	5	43--	65	8	28	7	7	6	54	10

-/--/++/+ low respectively high in comparison with the total group. Results are presented in percentages. Std = standard deviation. ? = question mark option use.
